# Gene expression analysis of cell death induction by Taurolidine in different malignant cell lines

**DOI:** 10.1186/1471-2407-10-595

**Published:** 2010-10-30

**Authors:** Ansgar M Chromik, Stephan A Hahn, Adrien Daigeler, Annegret Flier, Daniel Bulut, Christina May, Kamran Harati, Jan Roschinsky, Dominique Sülberg, Dirk Weyhe, Ulrich Mittelkötter, Waldemar Uhl

**Affiliations:** 1 Department of Visceral and General Surgery, St. Josef Hospital, Ruhr-University Bochum, Gudrunstrasse 56, D-44791 Bochum, Germany; 2 Department of Molecular Gastrointestinal Oncology, Ruhr-University Bochum, Universitätsstraße 150, D-44780 Bochum, Germany; 3 Department of Hand, Plastic and Reconstructive Surgery, Burn Center, BG-Unfallkrankenhaus, Ludwigshafen, Ludwig-Guttmann-Straße 13, D-67071 Ludwigshafen, Germany; 4 Department of Medicine I, St. Josef Hospital, Ruhr-University Bochum, Gudrunstrasse 56, D-44791 Bochum, Germany

## Abstract

**Background:**

The anti-infective agent Taurolidine (TRD) has been shown to have cell death inducing properties, but the mechanism of its action is largely unknown. The aim of this study was to identify potential common target genes modulated at the transcriptional level following TRD treatment in tumour cell lines originating from different cancer types.

**Methods:**

Five different malignant cell lines (HT29, Chang Liver, HT1080, AsPC-1 and BxPC-3) were incubated with TRD (100 μM, 250 μM and 1000 μM). Proliferation after 8 h and cell viability after 24 h were analyzed by BrdU assay and FACS analysis, respectively. Gene expression analyses were carried out using the *Agilent *-microarray platform to indentify genes which displayed conjoint regulation following the addition of TRD in all cell lines. Candidate genes were subjected to *Ingenuity Pathways Analysis *and selected genes were validated by qRT-PCR and Western Blot.

**Results:**

TRD 250 μM caused a significant inhibition of proliferation as well as apoptotic cell death in all cell lines. Among cell death associated genes with the strongest regulation in gene expression, we identified pro-apoptotic transcription factors (EGR1, ATF3) as well as genes involved in the ER stress response (PPP1R15A), in ubiquitination (TRAF6) and mitochondrial apoptotic pathways (PMAIP1).

**Conclusions:**

This is the first conjoint analysis of potential target genes of TRD which was performed simultaneously in different malignant cell lines. The results indicate that TRD might be involved in different signal transduction pathways leading to apoptosis.

## Background

Taurolidine (TRD) - a derivate of the aminosulfoacid Taurin - has been clinically used for many years in peritonitis and catheter related blood stream infections due to its anti-microbial and anti-inflammatory properties [1-3]. Recently it has been shown, that TRD also exerts anti-proliferative and anti-neoplastic activity *in vitro *as well as *in vivo *[4,5]. TRD has been reported to inhibit proliferation and to induce programmed cell death in a variety of cell lines derived from malignant tumours e.g. glioblastoma [6,7], melanoma [8,9], mesothelioma [10,11], colon carcinoma [12,13], squamous cell oesophageal carcinoma [14] and sarcoma [15,16]. Recently, favourable pharmacokinetic and safety data for TRD have been reported following systemic application in healthy volunteers [17] as well as in patients with locally advanced gastric carcinoma and glioblastoma [18-20].

However, cell death inducing mechanisms of TRD remain to be fully elucidated. Both the mitochondrial dependent pathway [7,10,21-23] as well as the death receptor associated pathways have been reported for TRD [24,25,16,14]. Since the majority of information about TRD effects is provided from studies with one single cell line, there is a lack of a comprehensive view across several cell lines of different malignancies. So far, only two publications have addressed the changes in gene expression following TRD exposure to malignant cells using cDNA microarray techniques [14,16]. The aim of this study was therefore, to analyse gene expression by microarray analyses simultaneously in different malignant cell lines - to identify potential TRD target genes which displayed conjoint regulation in all cell lines.

## Methods

### Cell lines and culture conditions

Five different human neoplastic cancer cell lines were used for this experiment: HT29 colon carcinoma (CLS Cell Lines Service, Eppelheim, Germany), Chang Liver (HeLa contaminant, CLS Cell Lines Service, Eppelheim, Germany), HT1080 fibrosarcoma (ATCC - LGC Standards GmbH, Wesel, Germany), AsPC-1 pancreas carcinoma (CLS Cell Lines Service, Eppelheim, Germany) and BxPC-3 pancreas carcinoma (ATCC - LGC Standards GmbH, Wesel, Germany). Chang Liver cells were maintained with Dulbecco's Modified Eagle Medium (DMEM) - Hams's F12, whereas HT1080 cells were cultured in modified Eagle's medium (MEM). The remaining cell lines (HT29, AsPC-1, BxPC-3) were maintained in RPMI 1640 (Biowest, Nuaille, France). All cultures were supplemented with 10% fetal bovine serum, supplemented with penicillin (100 U/ml), streptomycin (100 μg/ml) and 2 mM L-Glutamine (Biowest, Nuaille, France). AsPC-1 and HT1080 cells were further supplemented with 1 mM Sodium Pyruvate. Cells were grown as subconfluent monolayer and cultured in 25 cm^2 ^flasks at 37°C and 5% CO_2 _in a humidified atmosphere.

### Reagents

TRD (Taurolin^®^) ultrapure powder (kindly provided by Geistlich Pharma AG, Wolhusen, Switzerland) was dissolved in a Povidon 5% solution (K16 Povidon, generously provided by Geistlich Pharma AG, Wolhusen, Switzerland) and sterile filtered to achieve the respective TRD concentrations. A Povidon 5% solution in equal volume served as a control for TRD treatment.

### BrdU proliferation assay

Cells were seeded to a density of 3 × 10^6 ^cells/well in 6-well plates (growth area 9.6 cm^2^/well) and incubated for 18-24 hours under the above mentioned culture conditions to obtain a subconfluent monolayer. Subsequently, cells were washed and incubated for another 2 hours before reagents were added to the culture medium. To examine the dose-response of TRD regarding its anti-proliferative activity, cells were incubated with increasing concentrations of TRD (100 μM, 250 μM, and 1000 μM) and Povidon 5% as control for 8 h and submitted to BrdU proliferation assay (5-bromo-2'-deoxyuridine)-ELISA (Roche Applied Science, Mannheim, Germany) according to the manufacturer's instructions. Our group has recently shown, that the TRD concentration range applied in this experiment (100 μM, 250 μM and 1000 μM) is suitable for examining anti-neoplastic activity in these five cell lines [26]. Based on the incorporation of the thymidine analogue BrdU during DNA synthesis, the amount of newly synthesised DNA and thus of proliferation cells is detected using a microplate absorbance reader Sunrise™ (Tecan trading AG, Switzerland) after applying anti-BrdU conjugated with peroxidase and enhancing a specific substrate reaction. BrdU assays were performed with 8 replicates of three independent experiments with consecutive passages. The incubation time of 8 h has been shwon to be appropriate for the BrdU proliferation assay in previous experiments (data not shown).

### Flow Cytometry Analysis

All five cell lines were incubated for 24 h with TRD 250 μM and Povidon 5% as control and submitted to FACS analysis. Our group has recently shown, that TRD 250 μM is sufficient to induce cell death in the respective cell lines [26]. However, for the current study, FACS analysis for this particular concentration (250 μM) was performed in 9-11 independent experiments with 4-5 consecutive passages. In brief, floating cells were collected together with the supernatant and adherent cells were harvested by trypsinisation. Cells were sedimented by centrifugation, resuspended and fixed in 195 μl binding buffer (Bender MedSystems, Vienna, Austria). Cell density in the cell suspension was adjusted to 2 × 10^3 ^cells/μl. Subsequently, 5 μl Annexin V-FITC (BD Biosciences, Heidelberg, Germany) was added to the cell suspension followed by gently vortexing and incubation for 10 min at room temperature in the dark. Thereafter, the cell suspension was centrifuged followed by resuspension in 190 μl binding buffer before 10 μl Propidiumiodide (Bender MedSystems, Vienna, Austria) was added. Cells were analyzed immediately using a FACS (fluorescence activated cell sorting) flow cytometer (FACS Calibur BD Biosciences, Heidelberg, Germany) for Annexin V-FITC and PI binding. For each measurement, 20.000 cells were counted. Dot plots and histograms were analyzed by CellQuest Pro software (BD Biosciences, Heidelberg, Germany). Annexin V positive cells were considered apoptotic; Annexin V and PI positive cells were identified as necrotic. Annexin V and PI negative cells were termed viable. All experiments were repeated 9-11 times with 4-5 consecutive passages. The incubation time of 24 h has been shwon to be appropriate for the FACS analysis in previous experiments lines [26].

### cDNA microarray analysis

Total RNA was purified from the cells after incubation with the different substances (Povidon 5%; TRD 100 μM, 250 μM and 1000 μM) for 6 h using the RNeasy KIT from Qiagen (Hilden, Germany), as specified by the manufacturer. RNA integrity was assessed using standard denaturing agarose gel electrophoresis. For microarray analyses, we used the Agilent Array platform employing the manufacturer's standard protocols for sample preparation and microarray hybridization. Briefly, total RNA (500 ng) from each sample was amplified and transcribed into fluorescent cRNA following the manufacturer's Quick Amp Labeling protocol (Version 5.7, Agilent Technologies). Since we aimed at an intensity-based analysis of the two-colour microarrays as suggested by 't Hoen et al. [27], RNA samples from individual series of analyses comprising of RNAs derived from one specific cell line under the various TRD treatments and control conditions (Povidon 5%) were either Cy-5 (HT29, Chang Liver and HT1080) or Cy-3 (BxPC3, AsPC1) labelled. Labelled samples were hybridized towards the Whole Genome Oligo Array (4 × 44 Kk, product no. G4412T; Agilent Technologies) and following the washing steps the arrays were scanned using the Agilent Scanner G2505B. Agilent's Feature Extraction software 10.7.3.1 (Feature Extraction Protocol GE2_ 105-Dec08) was used to analyze acquired array images. Subsequent data processing was performed using the GeneSpringGX11.01 software package (Agilent Technologies). Following quantile normalization of the raw data, data sets were further reduced by filtering the raw signal intensity value to ≥50 in at least one out of the 20 samples analyzed. A one-way ANOVA model followed by Tukey's HSD (Honestly Significant Difference) test was used to test the hypothesis that there was no difference in expression between the TRD treatment group and the Povidon control group and multiplicity correction (Benjamini-Hochberg) was included to control the false discovery rate (FDR) at 0.05%. In a pair-wise comparison for differentially expressed genes identified between POV and TRD 250 μM treated cell lines by ANOVA a subset of genes was identified that displayed a conjoint regulation in all five cell lines after TRD 250 μM treatment with a mean increase or decrease ≥ 2 fold compared to control treatment (Povidon 5%). This subset of genes was subjected to Ingenuity Pathways Analysis (Ingenuity^® ^Systems, Redwood City, CA, USA). Genes associated with biological functions in Ingenuity's Knowledge Base were considered for the analysis. Right-tailed Fisher's exact test was used to calculate a p-value determining the probability that each biological function assigned to that data set is due to chance alone.

### Real-time PCR for microarray data validation

Microarray data validation was performed for the selected candidate genes ATF3, GADD34, TRAF6, PMAIP1 as well as EGR1 for TRD 250 μM and Povidon 5% as control. Total RNA (2 μg) was reverse transcribed using the High Capacity cDNA Archive Kit (Applied Biosystems). RNA isolation was performed from cells harvested after 6 h following treatment. cDNA was synthesized using 2 μg of total RNA, oligo(dT)_18 _primers and 200 U of MMLV reverse transcriptase (Promega, Mannheim, Germany) following the manufacturer's protocol. qRT-PCR was performed using a SYBR Green I reaction mixture containing 75 mM Tris-HCl (pH 8.8), 20 mM ammonium sulfate, 0.01% (v/v) Tween 20, 2 mM magnesium chloride (all Sigma-Aldrich, Munich, Germany), 1 μl of a 600-fold dilution of SYBR Green I (BioWhittaker, Rockland, ME, USA), 2.5 U *Taq *polymerase (NEB, Frankfurt a.M., Germany), 0.2 mM dNTP (Promega, Mannheim, Germany) and 0.2 μM of forward and reverse primer (QIAgen, Hilden, Germany) in a final reaction volume of 20 μl. Reactions were run on a DNA Engine Opticon^®^2 cycler (MJ Research, Waltham, MA, USA). The cycling conditions consisted of 3 min initial denaturation at 94°C and 40 cycles of 94°C for 30 sec, 60°C for 30 sec, 72°C for 30 sec and 80°C for 3 sec. Fluorescence was measured at the last step of each cycle. Melting curves were obtained after each PCR run and showed single PCR products. cDNAs were run in duplicates, including non-RT (without reverse transcriptase) and no-template controls. PCR efficiencies were determined using serial dilutions of a cDNA derived from a cell line. Expression levels for genes of interest and for housekeeping genes were measured in independent PCR runs. Expression ratios were calculated as described by Pfaffl [28] including the geometric mean expression of the housekeeping genes GAPD and PPIA to normalize the expression data for the gene of interest. Primers used are available through the authors upon request.

### Western Blot

To validate the findings of changed gene expression on protein level, Western Blots were performed for selected target genes whenever appropriate antibodies were available. Total protein was purified from the cells after incubation for 8 h with Taurolidine 250 μM and Povidon 5% as control treatment. For this purpose floating cells were collected together with the supernatant and sedimented by centrifugation. After removal of the supernatant, adherent cells were added after harvesting with a cell scrapper and Cell Culture Lysis Reagent (Promega Corporation, Mannheim, Germany). Probes were incubated with a total amount of 50 μl Cell Culture Lysis Reagent (Promega Corporation, Mannheim, Germany) each for 1 h on ice. The cell remnants were sedimented by centrifugation leading to supernatants containing the purified protein. Protein concentration in each sample was determined using a Micro BCA protein assay kit (Pierce, Rockford, IL, USA). Aliquots that contained equal amount of total proteins from each sample were separated in SDS-polyacrylamide gels and electrophoretically transferred onto PVDF membranes (Bio-Rad Laboratories, Munich, Germany), which were subsequently probed for 1 h - 3 h at room temperature with ATF3 (C-19) or GADD34/PPP1R15A (S-20) antibodies (Santa Cruz Biotechnology, Heidelberg, Germany). Blots were further incubated for 1 h with secondary horseradish peroxidase (HRP)-conjugated anti-rabbit IgG (GE Healthcare, Little Chalfont, UK), and then developed using an enhanced chemiluminescence detection system (ECL Plus Western Blotting Detection System, GE Healthcare, Little Chalfont, UK) according to the manufacturers' instructions. After finishing the western blot for the respective target genes, each membrane was stripped with stripping buffer (100 mM NaOH, 2% SDS, 0.5% DTT, 1 h for 55C) and incubated with GAPDH antibody (FL-335) (Santa Cruz Biotechnology, Heidelberg, Germany) as loading control.

### Statistical analysis

Results of FACS-analysis (percentage of viable, apoptotic and necrotic cells) as well as results of BrdU assay (percentage of proliferating cells) are expressed as means ± SEM. Comparison between experimental groups (control, TRD 100 μM, TRD 250 μM and TRD 1000 μM) was performed using one-way ANOVA followed by Tukey's post-hoc test. P-values ≤ 0.05 were considered as statistically significant and indicated in the figures as follows: *** p ≤ 0.001, ** p ≤ 0.01, * p ≤ 0.05.

## Results

### TRD uniformly inhibits proliferation of all cell lines in a dose dependent manner

**Figure 1:**
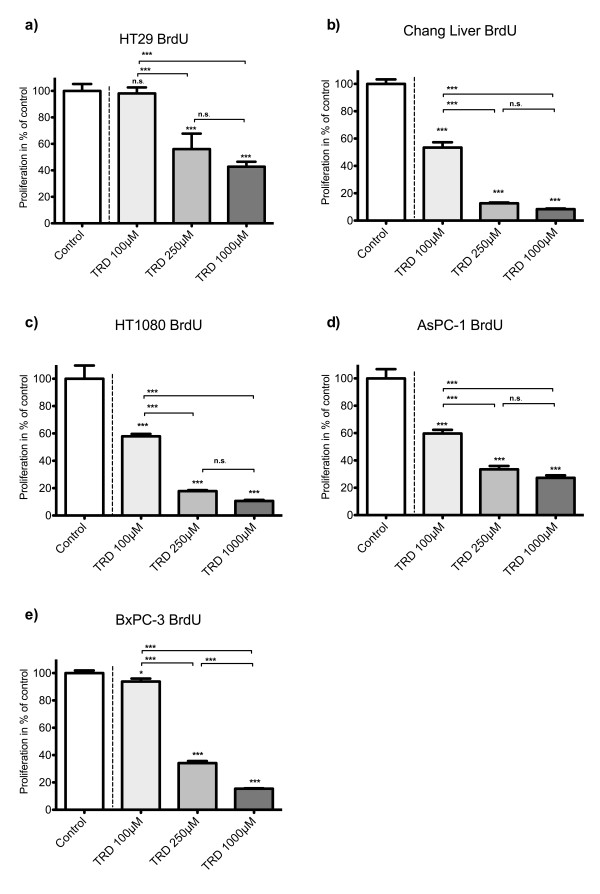
**Effects of Taurolidine (TRD) on proliferation in different malignant cell lines measured by BrdU-assay**. HT29 (a), Chang Liver (b), HT1080 (c), AsPC-1 (d) and BxPC-3 cells (e) were incubated with TRD (100 μM, 250 μM and 1000 μM) and with Povidon 5% (control) for 8 h and submitted to a BrdU-assay. Values are means ± SEM of 8 replicates of three independent experiments with consecutive passages. Asterisk symbols on columns indicate differences between control, which was set to 100% and TRD treatment. Asterisk symbols on brackets indicate differences between TRD groups. *** p ≤ 0.001, ** p ≤ 0.01, * p ≤ 0.05 (one-way ANOVA followed by Tukey's post-hoc test)

As indicated in figure 1, incubation with TRD in increasing concentrations (100 μM, 250 μM and 1000 μM) for 8 hours resulted in an dose dependent reduction of proliferating cells - as measured by BrdU assay. In all cell lines except HT29, even the lowest TRD concentration (100 μM) was capable of inhibiting proliferation leading to values of proliferating cells ranging between 53.4% (± 11.0%) (Chang Liver) and 93.8% (± 8.1%) (BxPC-3) which was significantly lower compared to untreated controls (100%) (Povidon 5%) (figure 1). However, the following TRD concentration of 250 μM significantly inhibited proliferation in all five cell lines. The pronounced inhibition mediated by TRD 250 μM resulted in values of proliferating cells between 12.7% (± 0.56%) for Chang liver and 56.1% (±11.7) for HT29 (figure 1a-b). The maximal dose of 1000 μM led to further inhibition of proliferation in all cell lines. As a result, dose response for cell proliferation was proportional in all cell lines (figure 1).

### TRD induces apoptotic cell death in all cell lines at a concentration of 250 μM

**Figure 2:**
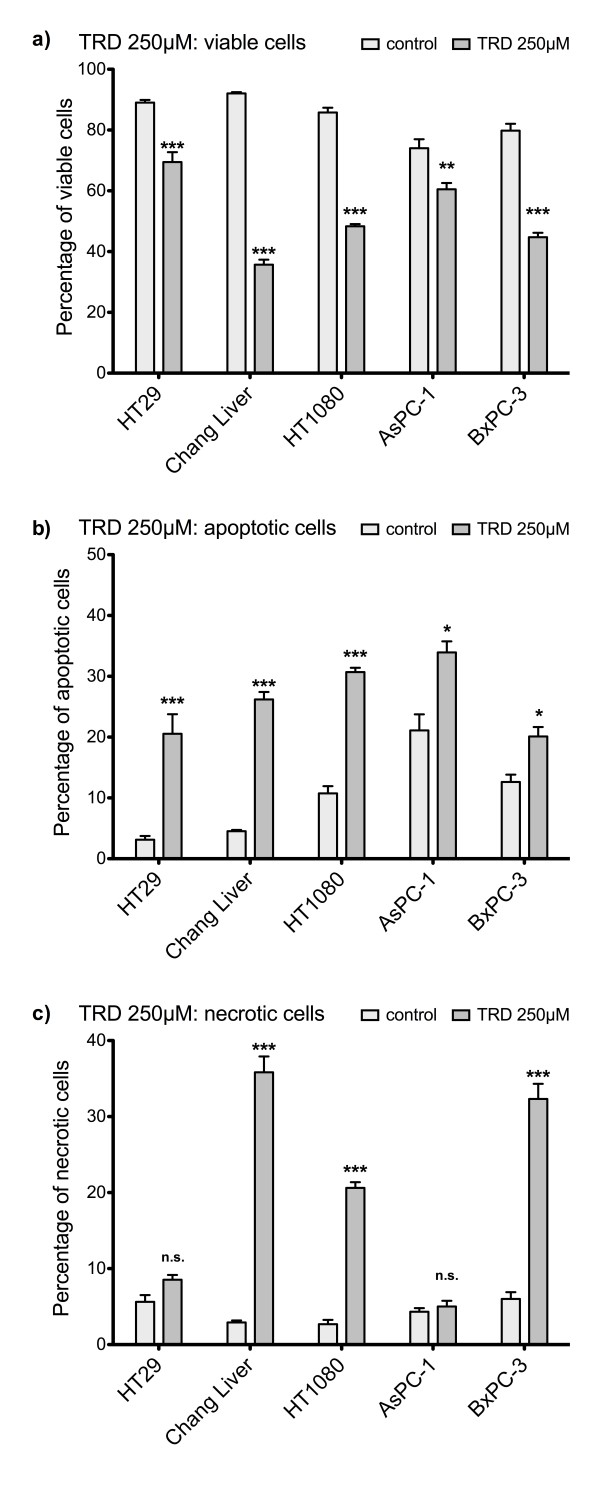
**Effects of Taurolidine (TRD) 250 μM on viability, apoptosis and necrosis in different malignant cell lines**. HT29, Chang Liver, HT1080, AsPC-1 and BxPC-3 cells were incubated with TRD 250 μM and with Povidon 5% (control) for 24 h. The percentages of viable (a), apoptotic (b) and necrotic cells (c) were determined by FACS-analysis for Annexin V-FITC and Propidiumiodide. Values are means ± SEM of 9 (HT29), 10 (Chang Liver, BxPC-3) or 11 (HT1080, AsPC-1) independent experiments with 4-5 consecutive passages. Asterisk symbols on columns indicate differences between control and TRD treatment. *** p ≤ 0.001, ** p ≤ 0.01, * p ≤ 0.05 (one-way ANOVA followed by Tukey's post-hoc test)

**Figure 3:**
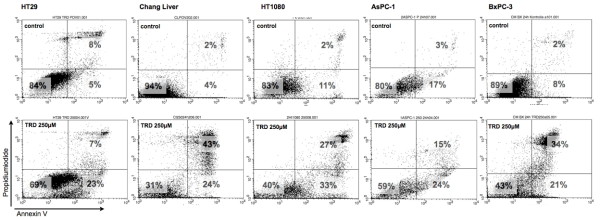
**Representative dot plots obtained by FACS analysis after incubation of different cell lines with Taurolidine**. HT29, Chang Liver, HT1080, AsPC-1 and BxPC-3 cells were incubated with Taurolidine (TRD) 250 μM and with Povidon 5% (control) for 24 h. FACS analysis was performed for Annexin V-FITC (x-axis) and Propidiumiodide (y-axis). Lower left quadrant: Annexin V and Propidiumiodide negative (viable cells), lower right quadrant: (Annexin V positive and Propidiumiodide negative (apoptotic cells), upper right quadrant: Annexin V and Propidiumiodide positive (necrotic cells).

As summarized in figure 2, incubation of the five cell lines with TRD 250 μM for 24 hours resulted in a significant reduction of viable cells compared to control treatment with Povidon 5% as evaluated by FACS analysis for Annexin V-FITC and PI. The TRD concentration of 250 μM was choosen in this experiment, since we could previously show that TRD 250 μM was the lowest among several concentrations (100 μM, 250 μM and 1000 μM) that significantly inhibited cell viability in all cell lines [26]. Furthermore, TRD 250 μM was the lowest concentration that significantly inhibited proliferation in all cell lines as measured by BrdU assay (figure 1). As shown in figure 2a, cell viability following incubation with TRD 250 μM was varying between 69.5% (± 3.2%) for HT29 and 35.7% (± 1.7%) for Chang Liver cells. The significant reduction of cell viability by TRD 250 M was paralleled by a significant increase of apoptotic cells in all cell lines (figure 2b). The apoptotic effect was ranging between 20.1% (± 1.5%) for BxPC-3 and 31.0% (± 2.4%) for AsPC-1) (figure 2b). Additionally, there was also a significant increase in necrosis in Chang Liver, HT1080 and BxPC-3 cells (figure 2c). Representative dot plots obtained by FACS analysis for incubation with TRD 250 μM and control treatment (Povidon 5%) in the five respective cell lines are presented in figure 3.

### TRD 250 μM leads to conjoint regulation of different genes in all five malignant cell lines

**Figure 4:**
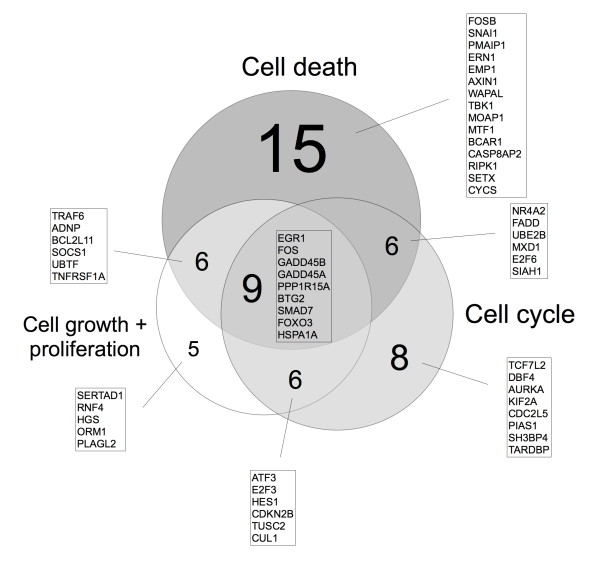
**Venn diagram for 55 genes identified by *Ingenuity Pathways Analysis^®^***. *Ingenuity Pathways Analysis *identified 55 Taurolidine regulated genes - in all five cell lines - to be involved in "*Cell death"*, "*Cell growth and proliferation" *and *"Cell cycle" *function. The 55 genes were significantly up- or down regulated in all five cell lines with a mean change ≥ 2 fold following Taurolidine (250 μM) treatment compared to control treatment (Povidon 5%). The Venn diagram illustrates the distribution and overlapping of Taurolidine regulated genes among the different functions.

In order to answer the question whether TRD treatment may modulate signalling pathways common in several different tumour types rather than being tumour cell type specific we subjected the above described different cell lines (HT29 (colon), Chang Liver (liver), HT1080 (fibrosarcoma), AsPC-1, BxPC-3 (both pancreas)) to global gene expression analyses using Agilent microarray technology. A comparison of the expression profiles in the TRD treatment (250 μM) vs. control treatment (Povidon 5%) revealed 592 probe sets that were significantly up- or down regulated in all five cell lines with a mean change ≥ 2 fold compared to the control treatment group (additional file 1, table S1). A total of 450 probe sets were significantly down-regulated in all cell lines with a mean decrease of 3.1 fold (95% CI 2.97 - 3.18) from control treatment to TRD 250 μM treatment. The remaining 142 genes were up-regulated with a mean increase of 7.3 fold (95% CI 6.97 - 7.72) from control treatment to TRD 250 μM treatment. From these genes we identified the top high-level functions by Ingenuity global function analysis (p < 0.05). 55 genes out of 592 probe sets were associated with at least one of the three functions "*Cell Death"*, "*Cell Growth and Proliferation"*, and *"Cell Cycle" *(figure 4). The function *"Cell Death" *was most frequent and associated with 36 genes, followed by "*Cell Cycle *(29 genes) and "*Cell Growth and Proliferation" *(29 genes) (figure 4) (additional file 1, table S1). The distribution and overlapping of target genes within the three functions is illustrated in figure 4. Potential TRD regulated genes common to all five cell lines that increased or decreased ≥ 4 fold and that were identified by *Ingenuity Pathways Analysis *are further summarized in table 1. There was a striking up-regulation of several transcription factors (TFs) like EGR1, ATF3, FOSB, FOS, SNAI1, NRA2 and HES1. Other groups of genes comprised cell cycle regulators (GADD45A, GADD45B, E2F3 and CDKN2B) as well as genes involved in the ubiquitin pathway (UBE2B, TRAF6), the endoplasmic reticulum response (PPP1R15A), the mitochondrial apoptotic pathway (PMAIP1) or the death receptor pathway (FADD) (table 1). The complete microarray data for all probe sets with the respective normalised values (all cell lines, Povidon 5%, TRD 100 μM, TRD 250 μM, TRD 1000 μM) are provided in additional file 2, table S2.

**Table 1:** Microarray results with characteristics of 21 genes regulated by Taurolidine identified by *Ingenuity pathway analysis*

Probe Set	Acession code	Gene symbol	Gene name	FC	Dir	Con	TRD	CD	CG	CC
A_23_P214080	NM_001964	EGR1	Homo sapiens early growth response 1	45,9	⇑	7,7	13,2	1	1	1
A_23_P34915	NM_004024	ATF3	Homo sapiens activating transcription factor 3	39,9	⇑	8,8	14,1	0	1	1
A_23_P429998	NM_006732	FOSB	FBJ murine osteosarcoma viral oncogene homolog B	38,7	⇑	5,8	11,0	1	0	0
A_23_P106194	NM_005252	FOS	v-fos FBJ murine osteosarcoma viral oncogene homolog	28,4	⇑	7,6	12,4	1	1	1
A_23_P131846	NM_005985	SNAI1	snail homolog 1	27,6	⇑	7,4	12,2	1	0	0
A_24_P239606	NM_015675	GADD45B	growth arrest and DNA-damage-inducible, beta	16,0	⇑	10,5	14,5	1	1	1
A_23_P207999	NM_021127	PMAIP1	phorbol-12-myristate-13-acetate-induced protein 1 = NOXA	10,2	⇑	8,0	11,3	1	0	0
A_23_P23221	NM_001924	GADD45A	growth arrest and DNA-damage-inducible, alpha	10,1	⇑	10,3	13,7	1	1	1
A_23_P131208	NM_006186	NR4A2	nuclear receptor subfamily 4, group A, member 2	9,2	⇑	5,3	9,0	1	0	1
A_23_P90172	NM_014330	PPP1R15A	protein phosphatase 1, regulatory (inhibitor) subunit 15A = GADD34	9,0	⇑	8,4	11,5	1	1	1
A_23_P436259	NM_152461	ERN1	endoplasmic reticulum to nucleus signalling 1 = IRE1	8,3	⇑	6,9	10,0	1	0	0
A_23_P218463	NM_013376	SERTAD1	SERTA domain containing 1	7,2	⇑	12,6	15,4	0	1	0
A_24_P921446	BC017854	EMP1	epithelial membrane protein 1	6,7	⇑	5,9	8,6	1	0	0
A_23_P385034	NM_001949	E2F3	E2F transcription factor 3	6,6	⇓	11,7	9,0	0	1	1
A_24_P278637	NM_003824	FADD	Fas associated death domain	5,9	⇓	9,3	6,8	1	0	1
A_23_P362415	BC001694	UBE2B	ubiquitin-conjugating enzyme E2B	5,7	⇑	4,5	7,0	1	0	1
A_24_P938293	NM_005524	HES1	hairy and enhancer of split 1	5,4	⇑	9,7	12,1	0	1	1
A_23_P75921	NM_145803	TRAF6	TNF receptor-associated factor 6	4,8	⇓	7,8	5,5	1	1	0
A_23_P408094	NM_002357	MXD1	MAX dimerization protein 1	4,7	⇑	6,7	8,9	1	0	1
A_23_P254179	NM_015339	ADNP	activity-dependent neuroprotector	4,3	⇓	10,8	8,7	1	1	0
A_24_P360674	NM_078487	CDKN2B	cyclin-dependent kinase inhibitor 2B	4,1	⇑	4,8	6,6	0	1	1

Microarray results with characteristics of 21 genes regulated by Taurolidine (TRD) 250 μM ≥ 4 fold compared to control treatment (Povidon 5%) which were identified by *Ingenuity pathway analysis*. **Abbreviations: **FC = fold change, Dir = direction of changed expression (⇑ = increased or ⇓ = decreased expression compared to control treatment), Con = mean normalised gene expression following control treatment (Povidon 5%), TRD = mean normalised gene expression following Taurolidine 250 μM treatment (raw = raw values, norm = normalised values), CD = cell death, CG = cell growth and proliferation, CC = cell cycle associated gene as analyzed by *Ingenuity pathway analysis *(1 = yes, 0 = no).

### Confirmation of altered gene expression following TRD 250 μM incubation by qRT-PCR and western blot

**Figure 5:**
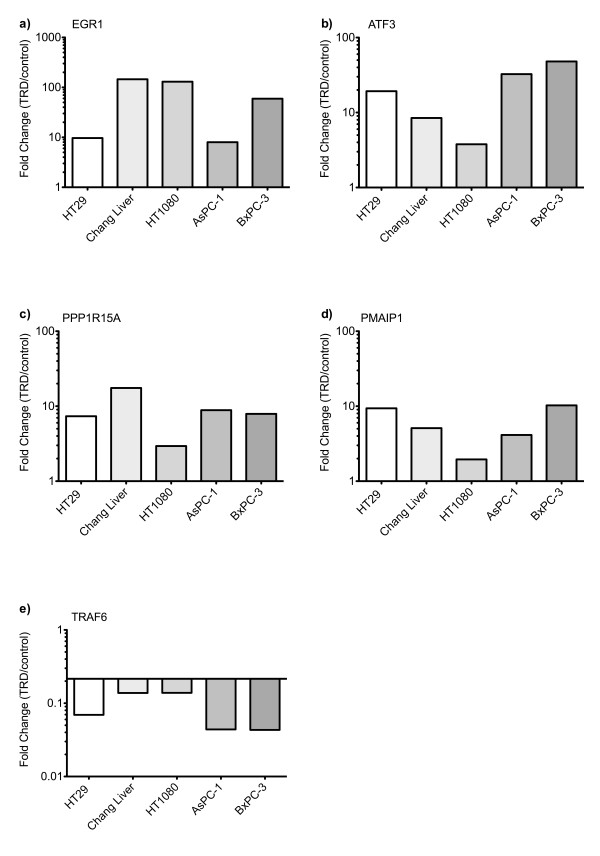
**Quantitative gene expression changes following Taurolidine treatment in five malignant cell lines**. HT29, Chang Liver, HT1080, AsPC-1 and BxPC-3 cells were incubated with Taurolidine (TRD) 250 μM and with Povidon 5% (control) for 6 h. For validation of altered gene expression observed during microarray experiments, relative expression levels compared to the control (5% Povidon) were determined by qRT-PCR for EGR1 (a), ATF3 (b), PPP1R15A (c), PMAIP1 (d) and TRAF6 (e).

**Figure 6:**
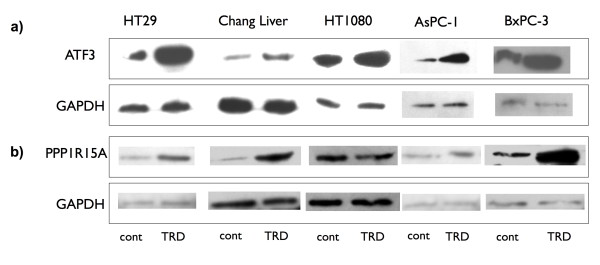
**Western blot analysis of selected genes targeted by Taurolidine treatment in five malignant cell lines**. HT29, Chang Liver, HT1080, AsPC-1 and BxPC-3 cells were incubated with Taurolidine (TRD) 250 μM and with Povidon 5% (control) for 8 h. Western blot analysis was performed for validation of altered gene expression observed during microarray experiments for ATF3 (a) and PPP1R15A (b). GAPDH served as loading control.

Messenger RNA expression of five selected TRD regulated genes was examined by quantitative real-time RT-PCR in all cell lines (figure 5). The gene with the strongest regulation during microarray analysis was EGR1 (Early growth response 1) with a mean fold change (FC) of 45.9 (table 1) which belongs to the EGR zinc-finger family of TFs [29] and is known to have anti-neoplastic activity [30]. This intensive induction could be confirmed by qRT-PCR, which revealed an up-regulation ranging from 8-fold to 145-fold (figure 5a). The second strongest regulation observed in the microarray analysis was referred to ATF3 (Activating transcription factor 3) (mean FC of 39.9) (table 1). ATF 3 is a member of the CREB family of transcription factors and is activated by various stimuli [31]. This result could also be confirmed by qRT-PCR, which resulted in a 4-fold to 48-fold increase (figure 5b). Two genes of special interest were also validated by qRT-PCR: PPP1R15A (Protein phosphatase 1, regulatory (inhibitory) subunit 15A; syn. GADD34), representing an important protein phosphatase involved in cell death pathways [32,33] as well as PMAIP1 (Phorbol-12-myristate-13-acetate-induced protein 1; syn. NOXA), a pro-apoptotic mitochondrial protein of the Bcl-2 family, which is known to be involved in the *intrinsic mitochondrial apoptotic pathway *[34]. Although the expressional changes in microarray experiments were lower than for the two other validated genes (mean FC of 9.0 and 10.2, respectively), comparable results could be obtained by qRT-PCR for PPP1R15A (3-fold to 18 fold increase) and PMAIP1 (2 fold to 10 fold increase) (figure 5c+d). TRAF6 (TNF receptor associated factor 6), a member of the E3 ubiquitin ligase family which can specificly target different proteins for Lys63 ubiquitination [35,36] was significantly down-regulated both in microarray analysis (mean FC of 4.8) and in qRT-PCR (5 to 9 fold decrease) (figure 5e). To further confirm altered gene expression of potential TRD target genes, western blots were performed when appropriate antibodies were available. As indicated in figure 6a, all cell lines displayed a pronounced upregulation of ATF3 protein, which was mirrored by the gene expression data as previously mentioned. PPP1R15A was also expressional regulated on protein level (figure 6b). However, in HT1080 cells we could not observe a convincing increase in PPP1R15A protein (figure 6b), which was also less pronounced on mRNA level (figure 5c).

## Discussion

Taurolidine (TRD) represents an interesting anti-neoplastic agent with a potential perspective in oncologic pharmacotherapy. Although it has already been intravenously applied to patients with advanced gastric cancer [18] and glioblastoma during pilot studies with promising results [19,18], the mechanisms of action remains to be elucidated in detail [4,5]. Microarray technology offers a powerful tool for investigating the cellular responses to anti-neoplastic substances like TRD because it monitors thousands of genes simultaneously. Although two recent publications from our group have already focussed on microarray derived transcriptional profiling of TRD treatment in two single cell lines [16,14], the current project provides the first microarray analysis which was performed simultaneously in five different and heterogeneous cell lines of four different malignancies to identify potential common TRD target genes.

Microarray experiments were focussed on a TRD concentration of 250 μM. We have recently shown in a study analysing TRD effects by FACS analysis in five cell lines identical with the current study (HT29, Chang Liver, HT1080, AsPC-1 and BxPC-3) that TRD inhibits cell viability over a broad range of concentrations (100 μM, 250 μM and 1000 μM) [26]. In the latter study, TRD 250 μM was the lowest concentration that significantly inhibited cell viability in all cell lines [26]. This fact is now supported by the current study, since TRD 250 μM significantly induced apoptotic cell death in our experiments - as measured by FACS analysis (figure 2+3). Furthermore, TRD 250 μM was the lowest concentration that significantly inhibited proliferation in all cell lines as measured by BrdU assay (figure 1). The effectiveness of TRD 250 μM as powerful anti-neoplastic concentration in cell culture experiments is consistent with several studies that evaluated the anti-proliferative and cell death inducing activity of TRD over a broad range of concentrations (50 μM - 2000 μM) in different cell lines using proliferation assays [37,11,39,10,16] or quantitative FACS analysis [16,14,13,11]. As indicated in figure 1 and 2, our results showed quite distinct and cell line specific differences in susceptibility towards TRD induced cell death. One can only speculate about the differences in the anti-neoplastic activity of TRD. However, none of the cell lines tested in our study was resistent to TRD induced cell death.

Using cRNA Microarray assay combined with *Ingenuity pathways analysis *we identified several TRD regulated genes that might represent potential target genes for TRD in malignant cells. Among the 21 genes with ≥ 4 fold expressional change (table 1), we focussed in our validation experiments on 5 genes, that represent - in our eyes - genes of special interest: the transcription factors (TFs) EGR1 and ATF3, the ER-stress response gene PPP1R15A, the pro-apoptotic mitochondrial protein PMAIP1 and the E3 ubiquitin ligase TRAF6. EGR1 (early growth response 1) representing the gene exhibiting the strongest induction upon TRD treatment was confirmed by qRT-PCR. EGR1 belongs to the EGR zinc-finger family of TFs [29] which can provide anti-neoplastic activity in numerous tumour cell lines by inducing tumour suppressors like PTEN, TGFβ1, p53, fibronectin or ATF3 [40,30,41]. Furthermore, several anti-neoplastic or chemopreventive agents have recently been shown to target EGR1 expression and EGR1 driven pathways in colorectal cancer [42-44]. Furthermore, our microarray analyses revealed a strong up-regulation of the pro-apoptotic transcription factor ATF3 (table 1), whereas other EGR1 target genes with tumour suppressor function like PTEN, TGFβ1, p53 and fibronectin did not show any significant regulation (additional file 2, table S2). The common induction of of EGR1 and ATF3 expression in all lines tested might indicate that activation of the EGR1/ATF3 signalling axis is an important mode of regulation how TRD mediates its growth suppressive action and/or apoptosis induction. The importance of EGR1/ATF3 axis in death signalling is supported by recent studies that described an EGR1 driven activation of the pro-apoptotic transcription factor ATF3 in colorectal cancer cells following treatment with anti-neoplastic substances [44,45]. ATF3 itself belongs to the CREB family of transcription factors and is regarded as a stress-inducible gene due to its activation by variety of stimuli e.g. radiation [31], chemotherapeutic agents [46,47] oxidative stress [48,49] or ER stress [50]. Although the physiologic functions of ATF3 are not well understood, there is some evidence that ATF3 functions as a pro-apoptotic gene in cancer cells. ATF3 has been shown to enhance the apoptotic effect of curcumin in squamous cell carcinoma cells [51] and to mediate nitric oxide-induced apoptosis in pancreatic beta cells [52]. Furthermore, ATF3 has been shown to promote UV induced apoptosis and cell cycle arrest in fibroblasts by binding the promoter and repressing the transcription of cyclin D1 -a key regulator of the G1-S checkpoint [53]. We can only speculate about the TRD driven stimulation of ATF3, but oxidative stress provides a possible pathway. Previous studies have presented first evidence for involvement of TRD mediated reactive oxygen species (ROS) production in malignant cells [7,25,26] and cell death induced by TRD has been shown to be reversible by application of radical scavengers like N-Acetylcysteine [7,54,25,10,26]. Another possible cell death associated pathway resulting in activation of ATF3 by TRD is the ER stress response pathway [55,56], since TRD has been reported to inhibit protein translation in tumour cells at an early stage [37]. Together with the above mentioned production of ROS, this might result in disturbance of ER homeostasis leading to ER stress response. Indicative of an involvement of the ER stress response pathway, we identified two genes that were up-regulated by TRD and known to be activated during ER stress, e.g. PPP1R15A (syn. GADD34) and the above mentioned ATF3. PPP1R15A represents an interesting potential TRD target gene, since PPP1R15A confers both apoptotic signalling [32,33] and inhibition of the translational silencing during ER stress via binding the catalytic subunit of protein phosphatase 1 leading to dephosphorylation of eIF2α [57,58]. Another strongly TRD regulated gene is PMAIP1 (syn. NOXA), a pro-apoptotic member of the BH3-only protein family, which is known to be involved in the *intrinsic mitochondrial apoptotic pathway *[34]. PMAIP1 exerts its pro-apoptotic function mainly by neutralizing anti-apoptotic members of the Bcl2 family through direct interaction, which has been proven for Bcl-2 related protein A1 (BCL2A1) and myeloid cell leukemia sequence 1 (MCL1) [59,60]. Interestingly, besides Bcl-2 like protein 11 (BCL2L11), which was 3-fold down-regulated, none of the remaining Bcl-2 family members (including MCL1 and BCL2A1) revealed a significant expressional change after TRD treatment in our experiments (additional file 2, table S2). Among the genes that were significantly down-regulated following TRD treatment, TRAF6 was considered as an especially important candidate gene. TRAF6 represents a member of the TRAF family of proteins that is involved in both the TNF receptor and the interleukin-1 receptor (IL-1R)/Toll-like receptor (TLR) superfamily signal transduction and is referred to innate and adaptive immunity, bone metabolism and developmental pathways [61,62]. Recently it has been shown that TRAF6 possesses an ubiquitin ligase activity domain (E3 ligase) that confers a site specific (Lys63) ubiquitination of different target proteins [35,36]. Interestingly, the protein kinase AKT - activated and transmitting survival signals in many tumours - has also been reported to be an ubiquitination target of TRAF6. Ubiquitination of AKT by TRAF6 is essential for its membrane recruitment and phosphorylation following growth factor activation [63]. Furthermore, inhibition of AKT phosphorylation has been described following TRD application in mesothelioma cells [11]. Although we did not find significant changes in the expression of AKT (additional file 2, table S2) and did not analyse AKT phosphorylation, the reduced expression of TRAF6 in our experiments provides a potential hint to the inhibition of the oncogenic AKT pathway by TRD. However, the involvement of the above mentioned pathways in TRD mediated cell death has to be addressed in further studies.

## Conclusions

This study provides the first identification of potential common target genes modulated by TRD treatment of cell lines originating from four different cancer types. Among the TRD regulated genes, we identified pro-apoptotic transcription factors, cell cycle regulators and proteins involved in ubiquitination, endoplasmic reticulum response and mitochondrial apoptotic pathways. Our results indicate that TRD is involved in different signal transduction pathways leading to multifaceted cell death mechanisms. Further studies are necessary to address the potential target genes in functional assays.

## Abbreviations

ATF3: Activating transcription factor 3; CDKN2B: Cyclin dependent kinase inhibitor 2B; E2F3: E2F transcription factor 3; EGR1: Early growth response 1; FC fold change; FOS: v-fos FBJ murine osteosarcoma viral oncogene homolog; FOSB: FBJ murine osteosarcoma viral oncogene homolog B; GADD34: Growth arrest and DNA-damage inducible 34; GADD45A: Growth arrest and DNA-damage inducible, alpha; GADD45B: Growth arrest and DNA-damage inducible, beta; HES1: Hairy and enhancer of split 1; NRA2: Nuclear receptor subfamily 4, group A, member 2; PI: Propidiumiodide; PMAIP1: Phorbol-12-myristate-13-acetate-induced protein 1 (Syn. NOXA); PPP1R15A: Protein phosphatase 1, regulatory (inhibitory) subunit 15A (Syn. GADD34); SNAI1: Snail homolog 1; TF: Transcription factor; TRAF6: TNF receptor-associated factor 6; TRD: Taurolidine; UBE2B: Ubiquitin-conjugating enzyme E2B.

## Competing interests

AMC received financial support by Geistlich Pharma (Suisse) for laboratory experiments. All other authors declare that they have no competing interests.

## Authors' contributions

AMC and SAH conceived of the study and its design, coordinated the experiments, carried out the statistical analysis and drafted the manuscript. AD and AF supervised the cell culture experiments and microarray experiments. DB was responsible for adjusting the FACS analysis and helped to draft the manuscript. CM, KH and JR carried out the cell culture experiments. DS helped with the statistical analysis and revised manuscript. DW, UM and WU participated in the design and coordination of the study and revised the manuscript. All authors have read and approved the final manuscript.

## Pre-publication history

The pre-publication history for this paper can be accessed here:

http://www.biomedcentral.com/1471-2407/10/595/prepub

## Supplementary Material

Additional file 1 **Probe sets that were significantly up- or down regulated in all five cell lines with a mean change ≥ 2 fold compared to control treatment**. Click here for file

Additional file 2 **All probe sets that were analysed in 5 malignant cell lines following incubation with various concentrations of Taurolidine (TRD) and Povidon 5% (Pov) as control treatment**. Click here for file
